# Exudative Retinitis in Bright’s Disease

**Published:** 1886-12-20

**Authors:** 


**Exudative Retinitis in Bright's Disease.** —Dr. E. Gruening, of New York, told a recent meeting of the Ophthalmological Society that he had collected over one hundred cases of this affection, and he found that none of them had lived over two years after the diagnosis of retinitis albuminuria had been made. In this class of cases he has included only those in which the typical stellate changes were seen in the maculæ of both eyes. He lately had seen this appearance in the macula of one eye in a patient who presents no evidence of Bright's disease. This was the first time he had seen this exquisite change without signs of renal disease.

